# Cancer Risk After Bariatric Surgery in a Cohort Study from the Five Nordic Countries

**DOI:** 10.1007/s11695-020-04751-6

**Published:** 2020-06-13

**Authors:** Wenjing Tao, Giola Santoni, My von Euler-Chelpin, Rickard Ljung, Elsebeth Lynge, Eero Pukkala, Eivind Ness-Jensen, Pål Romundstad, Laufey Tryggvadottir, Jesper Lagergren

**Affiliations:** 1 Upper Gastrointestinal Surgery, Department of Molecular Medicine and Surgery, Karolinska Institutet, Karolinska University Hospital, Retzius väg 13a, 171 77 Stockholm, Sweden; 2 Department of Public Health, University of Copenhagen, Copenhagen, Denmark; 3 Unit of Epidemiology, Institute of Environmental Medicine, Karolinska Institutet, Stockholm, Sweden; 4 Finnish Cancer Registry, Institute for Statistical and Epidemiological Cancer Research, Helsinki, Finland; 5 Faculty of Social Sciences, University of Tampere, Tampere, Finland; 6 Department of Public Health and Nursing, NTNU, Norwegian University of Science and Technology, Trondheim, Norway; 7 Icelandic Cancer Registry, Icelandic Cancer Society, Reykjavik, Iceland; 8 Faculty of Medicine, Laeknagardur, University of Iceland, Reykjavik, Iceland; 9 School of Cancer and Pharmaceutical Sciences, King’s College London, London, UK

**Keywords:** Obesity, Metabolic surgery, Neoplasm, Scandinavian and Nordic countries, Registries

## Abstract

**Purpose:**

Obesity increases the risk of several cancers, but the influence of bariatric surgery on the risk of individual obesity-related cancers is unclear. This study aimed to assess the impact of bariatric surgery on cancer risk in a multi-national setting.

**Materials and Methods:**

This cohort study included all adults with an obesity diagnosis identified from national patient registries in all Nordic countries (Denmark, Finland, Iceland, Norway and Sweden) from 1980 to 2012. Cancer risk in bariatric surgery patients was compared with non-operated patients with obesity. Multivariable Cox regression provided adjusted hazard ratios (HRs) with 95% confidence intervals (CIs). Age, sex, calendar year, country, length of follow-up, diabetes, chronic obstructive pulmonary disease and alcohol-related diseases were evaluated as confounders.

**Results:**

Among 482,572 participants with obesity, 49,096 underwent bariatric surgery. Bariatric surgery was followed by a decreased overall cancer risk in women (HR 0.86, 95% CI 0.80–0.92), but not in men (HR 0.98, 95% CI 0.95–1.01). The risk reduction was observed only within the first five post-operative years. Among specific tumours, HRs decreased for breast cancer (HR 0.81, 95% CI 0.69–0.95), endometrial cancer (HR 0.69, 95% CI 0.56–0.84) and non-Hodgkin lymphoma (HR 0.64, 95% CI 0.42–0.97) in female bariatric surgery patients, while the risk of kidney cancer increased in both sexes (HR 1.44, 95% CI 1.13–1.84).

**Conclusion:**

Bariatric surgery may decrease overall cancer risk in women within the first five years after surgery. This decrease may be explained by a decreased risk of breast and endometrial cancer and non-Hodgkin lymphoma in women.

## Introduction

Substantial evidence supports an association between obesity and cancer at thirteen sites, including oesophagus (adenocarcinoma), gastric cardia, colon, rectum, liver, gallbladder, pancreas, breast (postmenopausal), corpus uteri, ovary, kidney (renal cell), meningioma, thyroid and multiple myeloma [1]. There is also some evidence for male breast cancer, prostate cancer and non-Hodgkin lymphoma [1]. Several biological mechanisms, involving the endocrine, inflammatory and immune systems, link obesity to cancer development [2]. Bariatric surgery normally results in substantial and long-lasting weight loss and reduces obesity-related morbidity and mortality [3]. Available studies indicate a 30–50% overall decreased risk of cancer following bariatric surgery [4–11]. However, studies on bariatric surgery and specific cancer sites have provided inconsistent results, which might partly be a reflection of the limited number of cancer cases and length of follow-up in these studies. Some studies have suggested a decreased risk of endometrial cancer [7, 10–12] and postmenopausal breast cancer [9, 11, 12], while others have not [5].

This study aimed to assess the association between bariatric surgery and cancer risk using a large cohort with long and complete follow-up.

## Material and Methods

### Study Design

This study used the Nordic Obesity Surgery Cohort (NordOSCo), described in detail elsewhere [13], that includes all individuals aged 18 years or above with a documented obesity diagnosis in the National Patient Registry in Denmark, Finland, Iceland, Norway or Sweden. The diagnosis is documented by a physician in inpatient or outpatient specialised care, before automatic reporting to the National Patient Registry. The study period spanned from January 1, 1980, to December 31, 2012, with variation between countries depending on when codes for bariatric surgery became available in the National Patient Registries, i.e. 1996–2011 (Denmark), 1997–2012 (Finland), 1999–2012 (Iceland), 2007–2011 (Norway) and 1980–2012 (Sweden). This allowed up to 33 years of follow-up. Individuals who received a cancer diagnosis, other than non-melanoma skin cancer, before the obesity diagnosis were excluded. The study was approved by the Ethical Review Boards and Data Inspectorates from the participating countries, and a waiver of informed consent are granted for all registry-based studies.

### Exposure

The study exposure was *bariatric surgery*. Information on bariatric surgery in the National Patient Registries was ascertained using surgical codes based on the Nordic Medico-Statistical Committee Classification of Surgical Procedures (NOMESCO) [14]. The bariatric procedures consisted mainly of gastric bypass (majority), gastric banding and vertical banded gastroplasty. Sleeve gastrectomy was uncommon in the Nordic countries during the study period and is not identifiable due to the lack of a specific surgical code. Reporting by healthcare providers to the National Patient Registries is mandatory and informs reimbursement, which ensures high completeness. A validation study on bariatric surgery coding in the National Patient Registry in Sweden has also found high accuracy with an overall positive predictive value of 97% for these procedures [15].

### Outcomes

Cancer events were identified from the National Cancer Registries. Tumour topography was determined based on entity codes from the Cancer Statistics for the Nordic countries (NORDCAN) [16]. Due to variation in cancer coding practices between countries, the NORDCAN database validates and recodes information from all Nordic cancer registries to ensure uniformity and comparability [16]. The outcomes were *all cancers (excluding non-melanoma skin cancer)*, *obesity-related cancers* and *specific tumour sites* with a minimum of 20 cancer events in the bariatric surgery group. Obesity-related cancers included the thirteen tumours listed by the International Agency for Research on Cancer [1].

### Confounders

Eight variables that may be associated with both obesity and overall cancer risk were evaluated as potential confounders: age, sex, calendar year, country, length of follow-up, diabetes, chronic obstructive pulmonary disease representing tobacco smoking and alcohol dependency or alcohol-induced diagnoses representing alcohol overconsumption [17].

### Statistical Analysis

*Exposed* person-time was accumulated from the date of bariatric surgery until the date of any cancer, death, emigration or end of study period, whichever occurred first. *Unexposed* person-time was accumulated from the date of obesity diagnosis until the date of bariatric surgery, any cancer (excluding non-melanoma skin cancer), death, emigration or end of study period. Thus, bariatric surgery patients could contribute with both unexposed and exposed person-time. If a participant received an obesity diagnosis prior to the start of the study period, then person-time was accumulated from January 1st of the year that the country entered the cohort. Information on emigration and death was retrieved from the National Population Registries.

Cancer incidence in bariatric surgery patients was compared with non-operated individuals with obesity using multivariable Cox proportional hazards regression, providing hazard ratios (HRs) and 95% confidence intervals (CIs). The time scale was attained age. Data were split by date of bariatric surgery (time-varying exposure) and years of follow-up from the date of study entry (unexposed) or the date of bariatric surgery (exposed). The eight potential confounders were assessed through backward stepwise selection. If the likelihood ratio test was significant at the 5% level and the coefficient of the exposure changed by more than 2% (conservative value), the confounder was retained in the final model. Proxies for tobacco smoking and excess alcohol consumption did not improve model fit and were excluded from the final model. Thus, the multivariable Cox regression adjusted for sex (female or male), calendar year (continuous), country (Denmark, Finland, Iceland, Norway or Sweden), follow-up period (0–4 years, 5–9 years or ≥ 10 years) and diabetes (yes or no). To further understand the potential differences in prevalence of tobacco smoking between bariatric surgery patients and the obese comparison group, separate Cox regression analyses were undertaken to compare the incidence of lung cancer between the two groups.

The proportional hazards assumption was verified using Schoenfeld residuals. Because the assumption was not met for the single covariate sex, the model allowed different baseline hazard functions for men and women. Interactions between sex and the variables country, calendar period, follow-up period and diabetes were also included. To compute HRs for men and women separately, an interaction term was introduced between the variable sex and the exposure. To compute HRs at different follow-up periods, an interaction term was introduced between the variable for follow-up periods and the exposure variable, i.e. bariatric surgery. In stratified analyses, cohort participants were grouped into three follow-up periods after study entry or date of bariatric surgery: 0–4, 5–9 and ≥ 10 years. All statistical tests were two-sided and the significance level was 5%. The statistical software Stata/MP version 15.1 (StataCorp LLC, College Station, TX, USA) was used for all analyses.

## Results

### Participants

The cohort consisted of 482,572 individuals with obesity, including 49,096 who had undergone bariatric surgery. Censoring for death and emigration were 8.0% and 0.4%, respectively. Characteristics of the study participants are presented in Table 1. There were differences in age distribution and country of residence between operated and non-operated individuals.

**Table 1.** Characteristic of individuals with obesity in the study cohort (N=482,572), including 49,096 bariatric surgery patients.

**Characteristics**	**No bariatric surgery Number (%)**	**Bariatric surgery Number (%)**
**Age at entry (years)**		
<25	55,295 (11.7)	3,273 (6.7)
25-34	92,118 (19.5)	10,819 (22.0)
35-44	86,388 (18.3)	16,511 (33.6)
45-54	84,349 (17.9)	12,539 (25.5)
55-64	79,612 (16.9)	5,618 (11.4)
≥65	74,305 (15.7)	336 (0.7)
**Sex**		
Men	153,471 (32.5)	12,563 (25.6)
Women	318,596 (67.5)	36,533 (74.5)
**Country**		
Denmark	195,260 (41.4)	3,209 (6.5)
Finland	83,313 (17.7)	4,327 (8.8)
Iceland	14,539 (3.1)	716 (1.5)
Norway	32,189 (6.8)	5,374 (11.0)
Sweden	146,766 (31.1)	35,470 (72.3)
**Calendar period at entry**		
<1990	20,229 (4.3)	2,314 (4.7)
1990-1999	81,534 (17.3)	6,664 (13.6)
2000-2004	73,181 (15.5)	3,120 (6.4)
2005-2009	182,081 (38.6)	15,284 (31.1)
≥2010	115,042 (24.3)	21,714 (44.2)
**Follow-up time (years)**		
≤4	266,256 (56.4)	32,605 (66.4)
5-9	116,451 (24.7)	6,841 (13.9)
≥10	89,360 (18.9)	9,650 (19.7)
**Comorbidities**		
Chronic obstructive pulmonary disease	13,546 (3.0)	582 (1.2)
Diabetes	48,226 (10.2)	5,871 (12.0)
Excess alcohol consumption	13,245 (2.8)	1,088 (2.2)
**Bariatric surgery procedure**		
Gastric bypass	NA	35,541 (72.4)
Restrictive	NA	10,791 (22.0)
Other	NA	2,764 (5.6)
**Cancer events**		
All cancers^a^	24,565 (5.2)	1,314 (2.7)
Obesity-related cancers	12,789 (2.7)	706 (1.4)
Non-obesity related cancers	11,436 (2.4)	570 (1.2)
Specific cancers^b^		
Breast	3,328 (13.5)	179 (13.6)
Endometrium	2,050 (8.3)	113 (8.6)
Colon	2,066 (8.4)	99 (7.5)
Kidney	990 (4.0)	87 (6.6)
Rectum	913 (3.7)	45 (3.4)
Pancreas	808 (3.3)	41 (3.0)
Non-Hodgkin lymphoma	781 (3.2)	38 (2.9)
Leukaemia	499 (2.0)	32 (2.4)
Thyroid	300 (1.2)	20 (1.5)
Liver	617 (2.5)	18 (1.4)
Gallbladder	251 (1.0)	13 (1.0)
Multiple myeloma	259 (1.1)	11 (0.8)
Oesophagus	260 (1.1)	11 (0.8)

^a^Excluding non-melanoma skin cancer

^b^A person can have more than one cancer site registered at the same date of diagnosis.

### Risk of Cancer Overall and Obesity-Related Cancer

Compared with non-operated individuals with obesity, bariatric surgery patients had an 11% decreased overall risk of cancer (HR 0.89, 95% CI 0.83–0.94), and the risk was similarly reduced for obesity-related cancer (HR 0.89, 95% CI 0.82–0.97) (Table 2). The risk reduction was only found in women (HR 0.86, 95% CI 0.80–0.92) and not in men (HR 0.98, 95% CI 0.95–1.01), and the decreased risk was statistically significant only within the first five years of surgery (Table 2).

**Table 2.** Cancer risk comparing patients with bariatric surgery to individuals with obesity and without this surgery, presented as hazard ratio (HR) and 95% confidence interval (CI).

**Outcome**	**All**	**Men**	**Women**
	**Adjusted HR**^**a**^ **(95% CI)**	**Adjusted HR**^**a**^ **(95% CI)**	**Adjusted HR**^**a**^ **(95% CI)**
**All cancers**^**b**^			
**All years**	**0.89 (0.83-0.94)**	**0.98 (0.95-1.01)**	**0.86 (0.80-0.92)**
0-4 years	0.79 (0.72-0.87)	--	--
5-9 years	0.91 (0.80-1.03)	--	--
≥10 years	0.98 (0.89-1.07)	--	--
**Obesity-related cancers**			
**All years**	**0.89 (0.82-0.97)**	**1.04 (0.87-1.24)**	**0.86 (0.78-0.94)**
0-4 years	0.77 (0.67-0.88)	--	--
5-9 years	0.91 (0.76-1.09)	--	--
≥10 years	1.01 (0.90-1.13)	--	--
**Specific cancers**			
Colon	1.12 (0.90-1.39)	1.11 (0.78-1.59)	1.13 (0.86-1.48)
Rectum + anus	1.07 (0.77-1.48)	1.09 (0.67-1.76)	1.05 (0.68-1.63)
Pancreas	1.10 (0.78-1.57)	1.00 (0.53-1.87)	1.16 (0.76-1.77)
Breast	NA	NA	0.81 (0.69-0.95)
Endometrium	NA	NA	0.69 (0.56-0.84)
Kidney	1.44 (1.13-1.84)	1.53 (1.05-2.23)	1.39 (1.01-1.90)
Thyroid	0.72 (0.44-1.17)	0.21 (0.03-1.55)	0.84 (0.50-1.39)
Non-Hodgkin lymphoma	0.76 (0.54-1.07)	1.09 (0.63-1.88)	0.64 (0.42-0.97)
Leukaemia	1.04 (0.70-1.55)	1.16 (0.59-2.27)	0.99 (0.61-1.60)

^a^Adjusted for age, sex, country, calendar period, follow-up time, and diabetes.

^b^Excluding non-melanoma skin cancer

### Risk of Specific Cancer Types

Analyses of ten individual cancer sites with at least 20 cases in the bariatric surgery group indicated decreased risks of breast cancer (HR 0.81, 95% CI 0.69–0.95), endometrial cancer (HR 0.69, 95% CI 0.56–0.84) and non-Hodgkin lymphoma (HR 0.64, 95% CI 0.42–0.97) in women after bariatric surgery. The risk of kidney cancer was increased in both sexes following bariatric surgery (HR 1.44, 95% CI 1.13–1.84). No statistically significant associations were found between bariatric surgery and cancer of the colon, rectum, pancreas or thyroid or leukaemia (Table 2). Although not an obesity-related cancer, HRs were estimated for lung cancer to better understand smoking prevalence. The results showed that lung cancer risk was significantly decreased after bariatric surgery in men (HR = 0.53; 95% CI 0.31–0.89), but not in women (HR = 0.88; 95% CI 0.66–1.18).

## Discussion

This study indicates a slightly decreased risk of cancer after bariatric surgery, but the risk reduction attenuated five years after surgery. Reduced risks of breast cancer, endometrial cancer and non-Hodgkin lymphoma was observed for women, whereas the risk of kidney cancer was increased in both sexes.

This study with long and complete follow-up is based on one of the largest bariatric surgery cohorts to date, which has a sample size comparable to the accumulated number of cancer cases in meta-analyses on this topic [4–6]. This was enabled through inclusion of participants from all Nordic countries, which improves statistical power and facilitates generalisability. Because the Nordic populations have similar sociodemographic characteristics, genetic profiles, cultural traditions and norms and healthcare systems, the study population can be considered relatively homogenous. The nationwide coverage of the population and health data registries underlying this study reduces the risk of a biased selection of bariatric surgery patients, and the registries have repeatedly proven to include data of high validity [18–22].

Limitations to this study include selection bias of the non-operated participants with obesity. Because only individuals with a diagnosis of obesity obtained from inpatient care and outpatient specialised care were included in the cohort, they may have poorer health status than individuals with obesity in the general population. This possible excess comorbidity might be associated with an increased cancer risk. However, we accounted for several possible confounders, including age and comorbidities. Residual confounding might still apply because it was not possible to account for potential confounders that are not available from the registries, such as body mass index (BMI). It is not clear how the BMI of operated and non-operated participants differs in this study cohort, but a separate study based on a subset of the Swedish participants in the current cohort obtained pre- and post-operative BMI data; the mean preoperative BMI was 42.4 in patients who underwent bariatric surgery and 40.1 in individuals with obesity who did not undergo such surgery [23]. There was a mean weight loss of 20 to 30% within the initial two years of bariatric surgery in that cohort, and weight-loss remained at 15 to 25% ten years post-surgery. Gastric bypass resulted in the largest weight reduction, and the majority (72%) of bariatric surgery patients in the current study cohort underwent this procedure [24].

Tobacco smoking is a risk factor for several cancers [25]. Although smoking is not a contraindication for bariatric surgery, patients are often encouraged to give up smoking before surgery [26]. Recent studies have shown that up to half of the patients remain smokers on the day of surgery, and this proportion is probably higher for the current study cohort that includes patients from earlier time periods when smoking cessation before surgery was not widely promoted [28]. Chronic obstructive pulmonary disease as a proxy indicator for smoking did not improve model fit, indicating that smoking may not be a strong confounding factor in this study. In an explorative analysis, we found that lung cancer risk was decreased after bariatric surgery in men in the cohort, indicating that some of the lower cancer incidence observed in men might be due to lower rates of smoking. Yet, such confounding is unlikely to have changed the main results because the inverse associations between bariatric surgery and cancer risk were found mainly in women, where no difference in lung cancer was observed.

This study supports findings from meta-analyses and original studies that report a decreased overall risk of cancer following bariatric surgery [4–6], but the risk estimates in the current study indicate a weaker association than before that are mainly applicable to women. Meta-analyses examining separate tumour sites have found a decreased risk of endometrial cancer [28, 29] and breast cancer [30], after bariatric surgery, which is in line with the results of the present study. Bariatric surgery was also associated with a decreased risk of non-Hodgkin lymphoma, although the association was only found in women. Two smaller studies have indicated a decreased risk of non-Hodgkin lymphoma of similar magnitude as the current study, but the association was not statistically significant [6, 8]. The increased risk of kidney cancer after bariatric surgery was unexpected but confirms the finding from a recent British study [31]. The lack of a plausible mechanism, however, highlights the need for further research into this tumour.

The decreased cancer risk following bariatric surgery was only found during the initial five years after surgery. While limited statistical power in the longer follow-up periods might contribute to the lack of significant results, a possible explanation could be that weight loss, which is most substantial during the first post-operative years, has an independent protective effect on cancer. Recent studies from the Women’s Health Initiative indicate that intentional weight loss reduces the risk of obesity-related cancer independent of BMI [32] and that weight gain and weight cycling increase the risk of cancer irrespective of BMI [33]. It is also common that bariatric surgery patients regain weight with time after surgery, which might limit the protective effect of weight loss on cancer in the longer term [34]. Furthermore, the type of bariatric procedures performed have changed over time and might contribute to the differential results across follow-up periods; gastric bypass is currently the dominant procedure in the Nordic countries [13] and results in greater weight loss than restrictive procedures that were popular previously [34]. Previous studies have shown a stronger association between gastric bypass and reduced cancer risk than restrictive procedures [12].

Multiple biological mechanisms by which bariatric surgery reduces the risk of cancer have been proposed, including remission of insulin resistance and diabetes [35], lowered levels of bioactive sex steroids [36], modified levels of adipokins and decreased oxidative stress and systemic inflammation due to lesser adipose tissue [37]. Additional mechanisms might also apply for specific cancers. For example, bariatric surgery improves menstrual irregularity and polycystic ovarian syndrome that is a known risk factor for endometrial cancer [38, 39], and endometrial hyperplasia seems to resolve after bariatric surgery [40].

In conclusion, this large cohort study of all five Nordic countries indicates that the risk of obesity-related cancer is slightly reduced after bariatric surgery in women, but not in men, and that this reduction seems to attenuate over time after surgery. The decreased risk was particularly strong for breast cancer, endometrial cancer and non-Hodgkin lymphoma in women. A possible increased risk of kidney cancer merits further investigation.

## References

[1] Lauby-Secretan B, Scoccianti C, Loomis D, Grosse Y, Bianchini F, Straif K, International Agency for Research on Cancer handbook working G (2016). Body fatness and cancer--viewpoint of the IARC working group. N Engl J Med.

[2] Calle EE, Kaaks R (2004). Overweight, obesity and cancer: epidemiological evidence and proposed mechanisms. Nat Rev Cancer.

[3] Buchwald H, Avidor Y, Braunwald E, Jensen MD, Pories W, Fahrbach K, Schoelles K (2004). Bariatric surgery: a systematic review and meta-analysis. JAMA..

[4] Wiggins T, Antonowicz SS, Markar SR (2019). Cancer risk following bariatric surgery-systematic review and meta-analysis of national population-based cohort studies. Obes Surg.

[5] Tee MC, Cao Y, Warnock GL, Hu FB, Chavarro JE (2013). Effect of bariatric surgery on oncologic outcomes: a systematic review and meta-analysis. Surg Endosc.

[6] Casagrande DS, Rosa DD, Umpierre D, Sarmento RA, Rodrigues CG, Schaan BD (2014). Incidence of cancer following bariatric surgery: systematic review and meta-analysis. Obes Surg.

[7] Adams TD, Stroup AM, Gress RE, Adams KF, Calle EE, Smith SC, Halverson RC, Simper SC, Hopkins PN, Hunt SC (2009). Cancer incidence and mortality after gastric bypass surgery. Obesity..

[8] Sjostrom L, Gummesson A, Sjostrom CD, Narbro K, Peltonen M, Wedel H, Bengtsson C, Bouchard C, Carlsson B, Dahlgren S, Jacobson P, Karason K (2009). Effects of bariatric surgery on cancer incidence in obese patients in Sweden (Swedish Obese Subjects Study): a prospective, controlled intervention trial. Lancet Oncol.

[9] Christou NV, Lieberman M, Sampalis F, Sampalis JS (2008). Bariatric surgery reduces cancer risk in morbidly obese patients. Surg Obes Relat Dis.

[10] Anveden A, Taube M, Peltonen M, Jacobson P, Andersson-Assarsson JC, Sjoholm K, Svensson PA, Carlsson LMS (2017). Long-term incidence of female-specific cancer after bariatric surgery or usual care in the Swedish Obese Subjects Study. Gynecol Oncol.

[11] Schauer DP, Feigelson HS, Koebnick C, Caan B, Weinmann S, Leonard AC, Powers JD, Yenumula PR, Arterburn DE (2019). Bariatric surgery and the risk of cancer in a large multisite cohort. Ann Surg.

[12] Mackenzie H, Markar SR, Askari A, Faiz O, Hull M, Purkayastha S, Moller H, Lagergren J (2018). Obesity surgery and risk of cancer. Br J Surg.

[13] Tao W, Artama M, von Euler-Chelpin M, Konings P, Ljung R, Lynge E, Olafsdottir GH, Pukkala E, Romundstad P, Tryggvadottir L, Wahlin K, Lagergren J. Data resource profile: the Nordic obesity surgery cohort (NordOSCo). *Int J Epidemiol*. 2017;46:1367-67g. 10.1093/ije/dyx199 29040667

[14] Nordic Medico-Statistical Committee. NOMESCO classification of surgical procedures (NCSP), version 1.15. Copenhagen: Nordic Centre for Classifications in Health Care; 2009.

[15] Tao W, Holmberg D, Naslund E, Naslund I, Mattsson F, Lagergren J, Ljung R (2016). Validation of obesity surgery data in the Swedish National Patient Registry and Scandinavian Obesity Registry (SOReg). Obes Surg.

[16] Engholm G, Ferlay J, Christensen N, Bray F, Gjerstorff ML, Klint A, Kotlum JE, Olafsdottir E, Pukkala E, Storm HH (2010). NORDCAN--a Nordic tool for cancer information, planning, quality control and research. Acta Oncol.

[17] International Agency for Research on Cancer (2015). World cancer report 2014.

[18] Ludvigsson JF, Andersson E, Ekbom A, Feychting M, Kim JL, Reuterwall C, Heurgren M, Olausson PO (2011). External review and validation of the Swedish national inpatient register. BMC Public Health.

[19] Pukkala E, Engholm G, Hojsgaard Schmidt LK, Storm H, Khan S, Lambe M, Pettersson D, Olafsdottir E, Tryggvadottir L, Hakanen T, Malila N, Virtanen A (2018). Nordic cancer registries - an overview of their procedures and data comparability. Acta Oncol.

[20] Lynge E, Sandegaard JL, Rebolj M (2011). The Danish national patient register. Scand J Public Health.

[21] Bakken IJ, Gystad SO, Christensen OO, Huse UE, Laronningen S, Nygard J, Holmstrom L, Johannesen TB, Moller B, Larsen IK (2012). Comparison of data from the Norwegian patient register and the cancer registry of Norway. Tidsskr Nor Laegeforen.

[22] Sund R (2012). Quality of the Finnish hospital discharge register: a systematic review. Scand J Public Health.

[23] Sjöström L, Lindroos AK, Peltonen M, Torgerson J, Bouchard C, Carlsson B, Dahlgren S, Larsson B, Narbro K, Sjöström CD, Sullivan M, Wedel H, Swedish Obese Subjects Study Scientific Group (2004). Lifestyle, diabetes, and cardiovascular risk factors 10 years after bariatric surgery. N Engl J Med.

[24] Sjöström L (2013). Review of the key results from the Swedish obese subjects (SOS) trial - a prospective controlled intervention study of bariatric surgery. J Intern Med.

[25] World Cancer Research Fund (2007). Food, nutrition, physical activity and the prevention of cancer: a global perspective.

[26] Haskins IN, Nowacki AS, Khorgami Z, Schulz K, Heinberg LJ, Schauer PR, Brethauer SA, Aminian A (2017). Should recent smoking be a contraindication for sleeve gastrectomy?. Surg Obes Relat Dis.

[27] Gormsen J, Hjørne F, Helgstrand F. Cotinine test in evaluating smoking cessation at the day of bariatric surgery. Scand J Surg. 2019 [Epub ahead of print] 10.1177/1457496919866017 31342863

[28] Upala S, Anawin S (2015). Bariatric surgery and risk of postoperative endometrial cancer: a systematic review and meta-analysis. Surg Obes Relat Dis.

[29] Winder AA, Kularatna M, MacCormick AD (2018). Does bariatric surgery affect the incidence of endometrial cancer development? A systematic review. Obes Surg.

[30] Winder AA, Kularatna M, MacCormick AD (2017). Does bariatric surgery affect the incidence of breast cancer development? A systematic review. Obes Surg.

[31] Aravani A, Downing A, Thomas JD, Lagergren J, Morris EJA, Hull MA (2018). Obesity surgery and risk of colorectal and other obesity-related cancers: an English population-based cohort study. Cancer Epidemiol.

[32] Luo J, Hendryx M, Manson JE, Figueiredo JC, LeBlanc ES, Barrington W, Rohan TE, Howard BC, Reding K, Ho GY, Garcia DO, Chlebowski RT (2019). Intentional weight loss and obesity-related cancer risk. JNCI Cancer Spectr.

[33] Welti LM, Beavers DP, Caan BJ, Sangi-Haghpeykar H, Vitolins MZ, Beavers KM (2017). Weight fluctuation and cancer risk in postmenopausal women: the Women's Health Initiative. Cancer Epidemiol Biomark Prev.

[34] Sjostrom L (2012). Review of the key results from the Swedish obese subjects (SOS) trial: a prospective controlled intervention study of bariatric surgery. J Intern Med.

[35] Buchwald H, Estok R, Fahrbach K, Banel D, Jensen MD, Pories WJ, Bantle JP, Sledge I (2009). Weight and type 2 diabetes after bariatric surgery: systematic review and meta-analysis. Am J Med.

[36] Pugeat M, Crave JC, Elmidani M, Nicolas MH, Garoscio-Cholet M, Lejeune H, Dechaud H, Tourniaire J (1991). Pathophysiology of sex hormone binding globulin (SHBG): relation to insulin. J Steroid Biochem Mol Biol.

[37] Ashrafian H, Ahmed K, Rowland SP, Patel VM, Gooderham NJ, Holmes E, Darzi A, Athanasiou T (2011). Metabolic surgery and cancer: protective effects of bariatric procedures. Cancer..

[38] Sarwer DB, Spitzer JC, Wadden TA, Mitchell JE, Lancaster K, Courcoulas A, Gourash W, Rosen RC, Christian NJ (2014). Changes in sexual functioning and sex hormone levels in women following bariatric surgery. JAMA Surg.

[39] Skubleny D, Switzer NJ, Gill RS, Dykstra M, Shi X, Sagle MA, de Gara C, Birch DW, Karmali S (2016). The impact of bariatric surgery on polycystic ovary syndrome: a systematic review and meta-analysis. Obes Surg.

[40] Modesitt SC, Hallowell PT, Slack-Davis JK, Michalek RD, Atkins KA, Kelley SL, Arapovic S, Shupnik MA, Hoehn K (2015). Women at extreme risk for obesity-related carcinogenesis: baseline endometrial pathology and impact of bariatric surgery on weight, metabolic profiles and quality of life. Gynecol Oncol.

